# Association of dietary fat intake with skeletal muscle mass and muscle strength in adults aged 20–59: NHANES 2011–2014

**DOI:** 10.3389/fnut.2023.1325821

**Published:** 2024-01-17

**Authors:** Shijia Wang, Yu Zhang, Dandan Zhang, Fang Wang, Wei Wei, Qiong Wang, Yuanyuan Bao, Kang Yu

**Affiliations:** ^1^Department of Clinical Nutrition, The Second Affiliated Hospital of Soochow University, Suzhou, China; ^2^Department of Clinical Nutrition, Peking Union Medical College Hospital, Chinese Academy of Medical Sciences and Peking Union Medical College, Beijing, China; ^3^Department of Clinical Nutrition, Beijing Aerospace General Hospital, Beijing, China.

**Keywords:** fat, sarcopenia, appendicular lean mass, handgrip strength, non-aged, NHANES

## Abstract

**Background:**

Sarcopenia, a progressive loss of skeletal muscle mass and strength, needs to initially prevent in the twenties. Meanwhile, there is a lack of research on the effects of fat consumption on skeletal muscle mass and strength in adults aged 20–59. We aimed to assess associations between dietary fat intake and skeletal muscle mass, as measured by appendicular lean mass adjusted for body mass index (ALM_BMI_), and muscle strength, as represented by handgrip strength adjusted for body mass index (GSMAX_BMI_), among adults aged 20–59.

**Methods:**

Dietary fat intake per kilogram of actual body weight was assessed using two 24h recalls, while ALM and GSMAX were measured using DXA and a handgrip dynamometer, respectively. A weighted multiple linear regression model was employed to analyze the association between dietary fat intake and skeletal muscle mass, utilizing data from the National Health and Nutrition Examination Survey spanning from 2011 to 2014. To assess the non-linear relationship and saturation value between dietary fat intake and skeletal muscle mass, a smooth curve fitting approach and a saturation effect analysis model were utilized.

**Results:**

The study comprised a total of 5356 subjects. After adjusting for confounding factors, there was a positive association observed between dietary fat intake and ALM_BMI_ as well as GSMAX_BMI_. The relationship between dietary fat intake and ALM_BMI_ showed an inverted U-shaped curve, as did the association with GSMAX_BMI_. Turning points were observed at 1.88 g/kg/d for total fat intake and ALM_BMI_, as well as at 1.64 g/kg/d for total fat intake and GSMAX_BMI_. Furthermore, turning points were still evident when stratifying by gender, age, protein intake, and physical activity. The turning points were lower in individuals with low protein intake(<0.8 g/kg/d) and high levels of physical activity.

**Conclusion:**

The moderate dietary fat intake can be beneficial for muscle mass and strength in adults aged 20–59 under specific conditions. Special attention should be directed toward the consumption of fats in individuals with low protein intake and those engaged in high levels of physical activity.

## 1 Introduction

Sarcopenia, a condition characterized by the loss of skeletal muscle mass and strength, is associated with various negative outcomes such as falls, fractures, impaired daily activities, functional decline, frailty, and mortality (1, 2). It is estimated that the number of individuals affected by sarcopenia will rise from 50 million to more than 200 million worldwide in the next forty years (3). Additionally, sarcopenia poses a financial burden as it heightens the risk of hospitalization and increases the cost of care during hospital stays (4). While sarcopenia is widely acknowledged as an age-related condition (2), there are also numerous factors that contribute to its occurrence in early life (5). As the optimal levels of muscle mass and strength are typically achieved during the third and fourth decades of life (6, 7), it is advisable to commence proactive measures against sarcopenia in one’s twenties.

The Foundation of the National Institutes of Health (FNIH) Sarcopenia Project has successfully established clinically significant thresholds for identifying individuals with “low lean mass” (a decrease in skeletal muscle mass) and ‘weakness’ (reduced muscle strength). These thresholds were determined by assessing appendicular lean mass (ALM) or ALM adjusted for body mass index (BMI) [(ALM_*BMI)*_], as well as handgrip strength (GSMAX) or GSMAX adjusted for BMI (GSMAX_BMI’_) (8). These cut points represent distinct components of sarcopenia and have varying effects on human health.

In addition to aging, numerous factors, including disease processes, physical activity, diet, metabolic balance, and inflammation are associated with the decline in skeletal muscle mass and strength (9–11). As a modifiable factor, diet has been considered in the prevention and management of skeletal muscle mass and strength (12). Dietary fat intake has been found to potentially induce catabolic events in skeletal muscle and impact the differentiation of skeletal muscle stem cells (13, 14). Despite the limited research on the relationship between dietary fat intake and sarcopenia, particularly in young individuals, several studies have indicated that there may be a correlation between lower muscle mass and higher consumption of saturated fats (15), while monounsaturated fat and omega-3 fatty acid intake are linked to increased muscle strength in older individuals (16–18). However, the findings from other studies are not entirely consistent.

Consequently, the objective of our study was to examine the associations between dietary fat intake and markers of sarcopenia among adults aged 20–59 using data from the National Health and Nutrition Examination Survey (NHANES) 2011–2014. The variate outcomes of ALM_BMI_ and GSMAX_BMI_ were examined to eliminate the influence of body size on ALM and GSMAX. Likewise, the measurement of dietary fat intake was standardized as grams(g) or milligram (mg) per kilogram of body weight to control for the effect of body size.

## 2 Materials and methods

### 2.1 Data source and study population

**FIGURE 1:**
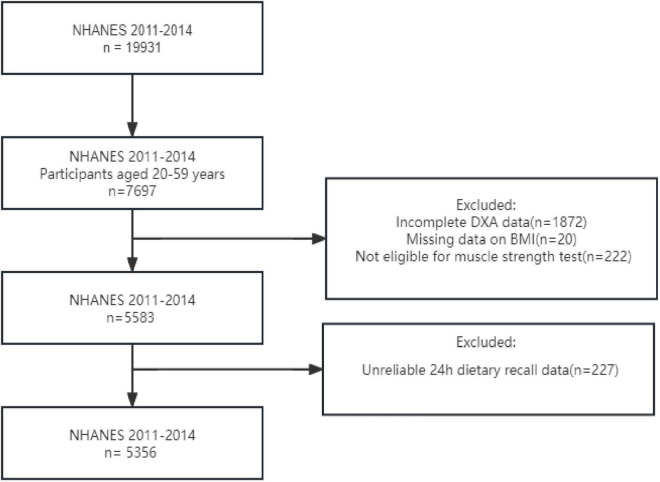
Flowchart of the participants selection.

This is a cross-sectional study data from a sub-sample of the NHANES from 2011 to 2014. NHANES is a survey conducted by the National Center for Health Statistics (NCHS) to evaluate health and nutritional data from a multi-stage representative sample of non-institutionalized. The NHANES study utilized a stratified multistage probabilistic sampling method to select a representative sample of the civilian non-institutionalized US population, with the objective of assessing the health and nutritional status of the US population. Ethical approval for this study was granted by the National Center for Health Statistics Research Ethics Review Board, and informed consent was obtained from all participants. Our analysis included 5356 participants from NHANES 2011 to 2014, specifically focusing on individuals aged between 20 and 59 years (7,697 individuals). We excluded incomplete Dual-energy X-ray absorptiometry (DXA) data for 1872 individuals, as well as those with missing data on BMI (20 individuals) and grip strength (222 individuals), along with unreliable 24h dietary recall data (227 individuals). After conducting these screenings mentioned above, our final sample consisted of a total of 5,356 individuals (Figure 1).

### 2.2 Dietary fat intake

Dietary fat intakes were assessed through two 24h dietary recall interviews, one conducted in-person at the mobile examination center (MEC) and the other via telephone 3–10 days later. The mean value of the two recalls was utilized when two complete and reliable recalls were available, while a single recall was used when necessary. Our analysis considered total fat intake, ω-3 fatty acids, including linolenic acid (18:3), stearidonic acid (18:4), eicosatetraenoic acid (20:5), clupanodonic acid (22:5), and docosahexaenoic acid (22:6), as well as ω-6 fatty acids, encompassing linoleic acid (18:2) and arachidonic acid (20:4). The average daily intake of ω-3 and ω-6 fatty acids was determined based on Dietary Studies from the U.S. Department of Agriculture’s Dietary Research Food and Nutrition Database (19) and standardized as grams (g) or milligrams (mg) per kilogram of body weight.

### 2.3 Body composition

During a visit to MEC, data on body measurements and composition were collected. Trained technicians measured individuals’ height in centimeters and weight in kilograms, from which BMI was calculated and rounded to one decimal place. To assess skeletal muscle mass, Dual-energy X-ray Absorptiometry (DXA) was used to determine ALM in kilograms (20). ALM represents lean soft tissue mass, excluding bone mineral content, from both arms and legs. This measurement was only calculated for those who had complete data for all four measurements. ALM_BMI_ was then determined by dividing ALM by BMI. In accordance with FNIH recommendations, low lean mass is defined as <0.789 (men) or <0.512 (women) based on ALM _BMI_ or <19.75 kg (men) or <15.02 kg (women) based solely on ALM values (8).

### 2.4 Handgrip strength and weakness

Muscle strength was assessed as a proxy indicator through the use of a handgrip dynamometer in the MEC. The measurement process involved three trials for each hand, with a 1-min rest period between measurements on the same hand. Participants who had undergone hand or wrist surgery within the past 3 months were excluded from testing that particular hand. Individuals unable to grip the dynamometer with either hand were considered to have missing handgrip strength data. The maximum value obtained from either hand, denoted as GSMAX, was subjected to analysis similar to that performed on the FNIH sarcopenia project. The variable GSMAX_BMI_ was derived by dividing GSMAX by BMI. In accordance with the recommendations of FNIH, weakness was defined as <1.0 (men) and <0.56 (women) based on GSMAX_BMI_ or <26 kg (men) and <16 kg (women) based on GSMAX (8).

### 2.5 Covariates

The study assessed various continuous variables, including age, height, weight, body mass index, daily energy intake and daily protein intake. Additionally, categorical variables such as gender (male, female), race (Mexican American, Non-Hispanic White, Non-Hispanic Black and Others), marital status (married/Living as married, separated and never married), income to poverty(≤1.3, >1.3, ≤3.5 and >3.5) (21), educational level(less than high school, high school or equivalent and college or above, smoking, drinking, hypertension, diabetes, coronary heart disease, cancer, physical activity(sedentary, low moderate and high) were considered. The classification of physical activity categories was determined using cut-points provided by established guidelines, which advocate for adults to engage in 500 to 1000 MET-minutes per week (22). The NHANES dataset offers methods for acquiring other covariate data.

### 2.6 Statistical analysis

The EmpowerStats 4.1 and R (4.2.3 version) software were employed for conducting statistical analysis in this study. Statistical significance was determined by considering a *p*-value below 0.05. All sample sizes were weighted in this investigation. Baseline data comparison involved presenting continuous variables as means ± standard deviation (SD) and *p*-value were computed using a weighted linear regression model. For categorical variables, *p*-value and percentages were obtained through chi-square testing. A weighted multiple linear regression analysis was conducted to examine the impact of fat consumption on variables ALM_BMI_ and GSMAX_BMI_. All confounding factors (age, gender, race, marital status, income to poverty, education level, health conditions and habits, physical activity, daily energy intake and daily protein intake) were taken into account when modeling. The purpose was to enhance the accuracy of reporting epidemiological observational studies and optimize the utilization of the collected data. Furthermore, the quartile methodology was employed to transform fat consumption into categorical group data, and the *p*-value for trend was calculated. Subgroup analyses were conducted to investigate the association between fat consumption and ALM_BMI_ and GSMAX_BMI_, while considering age, gender, physical activity and daily protein intake measured in grams per kilogram of body weight. Moreover, after adjusting for all potential confounding factors, weighted smooth curve fitting was performed, and a saturation effect analysis model was developed to evaluate the relationship. Results were expressed using turning point, effect-β (95%Cl, *p*-value), and the log-likelihood ratio test (LRT). They also stratified by age, gender, physical activity and daily protein intake as grams per kilogram of body weight and found turning point separately. The validity and correctness of all statistical analyses were ensured through rigorous verification by professional statisticians.

## 3 Results

### 3.1 Characteristics of participants

The characteristics of participants, categorized by quartile of total fat intake (g/kg/d), were presented in Table 1. In contrast to individuals in the highest quartile of total fat intake, those in the lowest quartile were found to be older, predominantly female, and exhibited a higher prevalence of weakness and sarcopenia. Additionally, a lower proportion of individuals in the lowest quartile reported current smoking and heavy drinking habits. Furthermore, individuals in the highest quartile of total fat intake demonstrated higher consumption of energy, carbohydrates, protein, and fiber compared to those in the lowest quartile.

**TABLE 1:** Characteristics of participants according to total fat intake NHANES, 2011—2014.

	Quartile 1	Quartile 2	Quartile 3	Quartile 4	*p*-value
**Demographic**					
Age, years	39.7 ± 11.5	40.3 ± 11.7	40.1 ± 11.7	37.1 ± 12.1	<0.0001
Gender, %					<0.0001
Male	40.5	50.6	53	60.4	
Female	59.5	49.4	47	39.6	
Race, %					0.0022
Mexican American	9	8.9	9.5	11.5	
Non-Hispanic White	61.3	65.1	66.8	63.3	
Non-Hispanic Black	14.5	10.8	10.2	10.3	
Others	15.2	15.2	13.4	15	
Marital status, %					<0.0001
Married/Living as married	61.5	63.8	62.1	56.4	
Separated	14.9	13.5	14	12.5	
Never married	23.6	22.7	23.9	31.2	
Income to poverty%					<0.0001
≤1.3	28.7	22.9	20.1	28.2	
>1.3, ≤3.5	36.5	32.6	30.8	34.5	
>3.5	34.8	44.6	49.1	37.3	
Education level					<0.0001
Less than high school	14.4	11.1	9.6	14.1	
High school or equivalent	21.9	18	17.9	19.6	
college or above	63.6	70.9	72.5	66.3	
**Health conditions and habits**					
Smoking, %					0.0016
Never	57.5	60.8	57.6	57.4	
Former	18.5	18.2	21.4	16.5	
Current	24	21.1	21.1	26.1	
Drinking, %					<0.0001
Non-drinker	18	14.5	11.4	14.2	
Low to moderate	68.9	70.1	69.8	67.8	
Heavy	13.1	15.4	18.7	18	
Hypertension, %					<0.0001
Yes	29.3	23	22.1	17.3	
No	70.7	77	77.9	82.7	
Diabetes, %					<0.0001
Yes	8.4	5	4.9	4	
No	88.7	93.8	92.8	94.6	
Borderline	2.9	1.2	2.2	1.4	
Don’t know	0.1	0	0	0	
**Coronary heart disease, %**					0.0549
Yes	1.3	1.3	1.3	0.4	
No	98.7	98.7	98.7	99.6	
Cancer, %					0.0499
Yes	6.1	6.2	4.8	4.2	
No	93.9	93.8	95.2	95.8	
**Physical activity, %**					<0.0001
Sedentary	20.2	17	16.6	16.1	
Low	37.6	42.7	40.7	34.5	
Moderate	15.9	15.7	16.2	16.3	
High	26.3	24.5	26.6	33	
**Anthropometrics and body composition**					
Weight, kg	91.2 ± 23.6	85.6 ± 19.1	80.1 ± 19.1	73.9 ± 16.3	<0.0001
Height, m	168.9 ± 9.9	169.6 ± 9.0	169.8 ± 9.4	170.3 ± 9.9	0.0018
BMI, kg/m	31.9 ± 7.6	29.7 ± 6.1	27.7 ± 5.8	25.4 ± 4.7	<0.0001
Waist	104.9 ± 17.3	100.2 ± 14.9	95.7 ± 15.0	89.8 ± 12.7	<0.0001
ALM, kg	23.7 ± 6.7	23.5 ± 6.0	22.9 ± 6.4	22.2 ± 6.0	<0.0001
ALM_BMI_	0.8 ± 0.2	0.8 ± 0.2	0.8 ± 0.2	0.9 ± 0.2	<0.0001
Low mean mass, %	17	13	17.5	19.5	<0.0001
**Muscle strength**					
Grip strength, kg	39.0 ± 11.4	40.6 ± 11.2	40.1 ± 11.1	41.2 ± 11.6	<0.0001
GSMAX_BMI_	1.3 ± 0.5	1.4 ± 0.4	1.5 ± 0.4	1.7 ± 0.5	<0.0001
Weakness, %	3.3	1.9	1.9	0.5	<0.0001
Sarcopenia, %	1.8	1.1	1.6	0.2	0.0007
**Dietary intake**					
Energy, kcal/day	1444.2 ± 486.1	1933.3 ± 494.9	2324.5 ± 598.5	3075.0 ± 897.0	<0.0001
Protein, g/day	58.9 ± 25.7	77.6 ± 26.9	90.3 ± 32.5	114.8 ± 41.6	<0.0001
Protein, g/kg/day	0.7 ± 0.3	0.9 ± 0.3	1.1 ± 0.4	1.6 ± 0.5	<0.0001
Carbohydrate, g/day	189.4 ± 74.4	237.4 ± 82.9	274.0 ± 90.0	351.6 ± 128.2	<0.0001
Fat, g/day	44.4 ± 16.3	69.3 ± 16.0	89.7 ± 22.7	129.2 ± 39.7	<0.0001
Fat, g/kg/day	0.5 ± 0.1	0.8 ± 0.1	1.1 ± 0.1	1.8 ± 0.5	<0.0001
Fat, %	28.2 ± 7.2	33.0 ± 6.3	35.3 ± 6.1	38.2 ± 6.3	<0.0001
Saturated fat, g/kg/day	0.2 ± 0.1	0.3 ± 0.1	0.4 ± 0.1	0.6 ± 0.2	<0.0001
Monounsaturated fat, g/kg/day	0.2 ± 0.1	0.3 ± 0.0	0.4 ± 0.1	0.6 ± 0.2	<0.0001
Polyunsaturated fat, g/kg/day	0.1 ± 0.0	0.2 ± 0.1	0.3 ± 0.1	0.4 ± 0.2	<0.0001
Total ω-6, mg/kg/day	102.5 ± 42.1	171.4 ± 47.6	234.2 ± 62.6	368.1 ± 140.7	<0.0001
Total ω-3, mg/kg/day	11.9 ± 6.1	19.6 ± 7.7	25.3 ± 9.7	39.7 ± 19.0	<0.0001
ALA,mg/kg/day	10.9 ± 5.5	18.3 ± 7.2	23.5 ± 8.5	37.5 ± 18.3	<0.0001
EPA,mg/kg/day	0.3 ± 0.7	0.3 ± 0.9	0.5 ± 1.3	0.5 ± 1.1	<0.0001
DHA,mg/kg/day	0.6 ± 1.5	0.7 ± 1.6	0.9 ± 2.5	1.0 ± 2.2	<0.0001
ω-6/ω-3 ratio	9.5 ± 4.2	9.4 ± 3.0	10.1 ± 4.2	10.0 ± 3.9	<0.0001
Fiber, g/day	12.1 ± 6.9	16.0 ± 8.3	18.9 ± 9.0	22.7 ± 10.4	<0.0001

BMI, body mass index; ALM_BMI_, appendicular lean mass adjusted for BMI; GSMAX_BMI_, handgrip strength adjusted for BMI; DHA, docosahexaenoic acid; EPA, eicosapentaenoic acid; ALA, alpha linolenic acid. Mean ± SD for: age, weight, height, BMI, waist, ALM_BMI_, appendicular lean mass, GSMAX_BMI_, low mean mass, GSMAX_*BMI*_, energy, protein, carbohydrate, fat, saturated fat, monounsaturated fat, polyunsaturated fat, total ω-6, total ω-3, ALA,EPA,DHA,ω-6/ω-3 ratio, fiber. *p*-value was calculated by weighted linear regression model. % for: age, gender, race, marital status, income to poverty, education level, health conditions and habits, Physical activity. *p*-value was calculated by weighted chi-square test.

### 3.2 Association of dietary fat intake with ALM_BMI_

**FIGURE 2:**
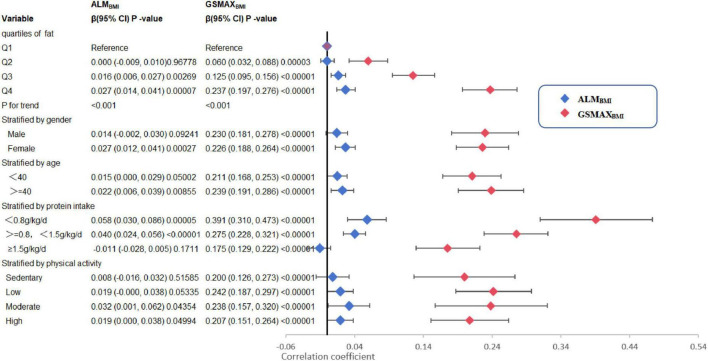
Trend analysis and stratified analysis for the association of total fat intake (g/kg/d) with ALM_BMI_ and GSMAX_BMI_, NHANES, 2011–2014. BMI, Body mass index; ALM_BMI_, appendicular lean mass adjusted for BMI; GSMAX_BMI_, handgrip strength adjusted for BMI. The model was the model 3 from Table 2. The model was not adjusted for the stratification variable itself in the subgroup analysis.

Three weighted univariate and multivariate linear regression models were developed: model 1, which was not adjusted; model 2, which adjusted for age, gender, and race; and model 3, which adjusted for age, gender, marital status, and income to poverty, education level, health conditions and habits, physical activity, energy and protein intake were adjusted. In model 3, a positive correlation was observed between total fat intake and ALM_BMI_ [β 0.018, 95% CI:(0.007, 0.029)]. Furthermore, saturated fat [(β 0.029, 95% CI:(0.002, 0.055)], monounsaturated fat [(β 0.046, 95% CI:(0.019, 0.072)], and polyunsaturated fat [(β 0.040, 95% CI:(0.009, 0.071)] all exhibited positive associations (Table 2). However, when stratifying the data by gender, age, protein intake, and physical activity, the association was found to be non-significant in males, individuals below 40 years of age, individuals with protein intake exceeding 1.5 g/kg/d and individuals with sedentary or low physical activity (Figure 2). When the quartile of total fat intake was constructed, the lowest quartile was used as a reference, the trend analysis was statistically significant (p for trend < 0.001), and the 4th quartile was significantly positively associated with ALM_BMI_, but the 2nd quartile was indifferent (Figure 2).

**TABLE 2:** Linear regression between dietary fat intake and ALM_BMI_ and GSMAX_BMI_, NHANES, 2011–2014.

	ALM_BMI_			GSMAX_BMI_		
	Model 1 β (95% CI)	Model 2 β (95% CI)	Model 3 β (95% CI)	Model 1 β (95% CI)	Model 2 β (95% CI)	Model 3 β (95% CI)
Fat, g/kg/day	0.084 (0.075, 0.094)***	0.042 (0.036, 0.047)***	0.018 (0.007, 0.029)***	0.261 (0.239, 0.284)***	0.173 (0.156, 0.189)***	0.224 (0.192, 0.255)***
Fat,%(energy)	−0.046 (−0.119, 0.026)	−0.006 (−0.046, 0.035)	−0.035 (−0.080, 0.010)	−0.317 (−0.488, −0.146)***	−0.208 (−0.331, −0.085)***	−0.296 (−0.431, −0.161)***
Saturated fat, g/kg/day	0.220 (0.194, 0.247)***	0.100 (0.085, 0.115)***	0.029 (0.002, 0.055)*	0.687 (0.625, 0.750)***	0.433 (0.387, 0.478)***	0.450 (0.372, 0.529)***
Monounsaturated fat, g/kg/day	0.232 (0.206, 0.258)***	0.113 (0.098, 0.127)***	0.046 (0.019, 0.072)***	0.691 (0.630, 0.752)***	0.447 (0.403, 0.492)***	0.460 (0.382, 0.539)***
Polyunsaturated fat, g/kg/day	0.234 (0.197, 0.271)***	0.139 (0.118, 0.160)***	0.040 (0.009, 0.071)*	0.759 (0.672, 0.846)***	0.560 (0.497, 0.622)***	0.466 (0.374, 0.558)***
Total ω-3, mg/kg/day	0.001 (0.001, 0.002)***	0.001 (0.001, 0.001)***	0.000 (−0.000, 0.000)	0.006 (0.005, 0.006)***	0.005 (0.004, 0.005)***	0.003 (0.002, 0.004)***
ALA,mg/kg/day	0.001 (0.001, 0.002)***	0.001 (0.001, 0.001)***	0.000 (−0.000, 0.000)	0.006 (0.005, 0.007)***	0.005 (0.004, 0.006)***	0.003 (0.003, 0.004)***
EPA,mg/kg/day	0.006 (0.001, 0.011)*	0.003 (−0.000, 0.006)	−0.003 (−0.006, 0.001)	0.022 (0.010, 0.034)***	0.017 (0.009, 0.026)***	−0.009 (−0.018, −0.000)*
DHA,mg/kg/day	0.003 (0.001, 0.006)*	0.003 (0.001, 0.004)***	−0.001 (−0.002, 0.001)	0.013 (0.006, 0.019)***	0.012 (0.008, 0.017)***	−0.006 (−0.011, −0.000)*
Totalω-6, mg/kg/day	0.0003 (0.0002, 0.0003)***	0.0002 (0.0001, 0.0002)***	0.0000 (0.0000, 0.0001)***	0.001 (0.001, 0.001)***	0.001 (0.001, 0.001)***	0.001 (0.000, 0.001)***
ω-6/ω-3 ratio	0.003 (0.002, 0.004)	0.001(−0.000, 0.001)	0.001 (0.000, 0.002)	0.004 (0.001, 0.008)	0.000 (−0.002, 0.003)	0.002 (−0.000, 0.005)

BMI, Body mass index; ALM_BMI_, appendicular lean mass adjusted for BMI; GSMAX_BMI_, handgrip strength adjusted for BMI; DHA, docosahexaenoic acid; EPA, eicosapentaenoic acid; ALA, alpha linolenic acid. Model 1, no covariates were adjusted. Model 2, age, gender and race were adjusted. Model 3, age, gender, race, marital status, income to poverty, education level, health conditions and habits, physical activity, energy and protein intake were adjusted. Values shown as coefficients and 95% confidence intervals (95%CI).

*p < 0.05,

**p < 0.01,

***p < 0.001.

**FIGURE 3:**
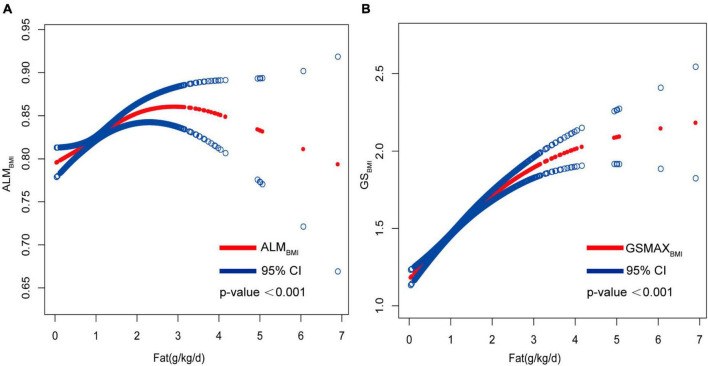
Correlation between total fat intake (g/kg/d) with ALM_BMI_ and GSMAX_BMI_. **(A)** Non-linear association between total fat intake (g/kg/d) with ALM_BMI_ and **(B)** non-linear association between total fat intake (g/kg/d) with GSMAX_BMI_. All confounding factors were adjusted.

**FIGURE 4:**
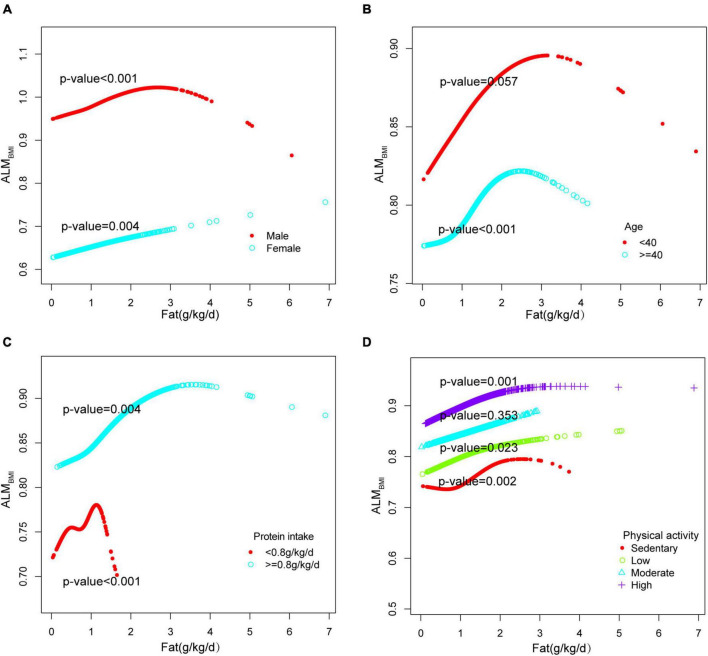
Stratified analysis for total fat intake (g/kg/d) and ALM_BMI_ dose–response relationship. **(A)** Stratified by sex **(B)** Stratified by age **(C)** Stratified by protein intake (g/kg/d). **(D)** Stratified by physical activity. confounding factors were adjusted. The model was not adjusted for the stratification variable itself in the subgroup analysis.

Adjusted smoothed plots suggested non-linear relationships between total fat intake and ALM_BMI_ (Figure 3A), stratified by gender, age, protein intake, and physical activity (Figure 4). ALM_BMI_ increased with total fat intake up to the inflection point (1.88 g/kg/day). Furthermore, significant inflection points were observed in individuals aged below 40 years old, as well as those with protein intake exceeding 0.8 g/kg/d, sedentary individuals, low levels of physical activity, or high levels of physical activity (Figure 4C and Table 3). Taken together, the association between total fat intake and ALM_BMI_ in the individuals above mentioned followed an inverted U-shaped curve. A M-shaped curve relationship was observed between total fat intake and ALM_BMI_ in the individuals with protein intake below 0.8 g/kg/d (Figure 4C), with significant inflection points at 0.43 g/kg/day and 1.07 g/kg/day (Table 3).

**TABLE 3:** Threshold effect analysis of fat intake on ALM_BMI_ and GSMAX_BMI_ using two-piece wise linear regression.

ALM_BMI_	Adjusted ß (95% CI), *p*-value	GSMAX_BMI_	Adjusted ß (95% CI), *p*-value
Inflection point	1.88	Inflection point	1.64
Fat < 1.88 g/kg/day	0.03 (0.02, 0.04) < 0.0001	Fat < 1.64 g/kg/day	0.28 (0.24, 0.31) < 0.0001
Fat > 1.88 g/kg/day	−0.01 (−0.03, 0.01) 0.3063	Fat > 1.64 g/kg/day	0.14 (0.09, 0.18) < 0.0001
log likelihood test	<0.001	log likelihood test	<0.001
**Protein intake**		**Protein intake**	
<0.8 g/kg/d		<0.8 g/kg/d	
Inflection point	0.43,1.07	Inflection point	1.01
Fat < 0.43 g/kg/day	0.20 (0.09, 0.31) 0.0006	Fat < 1.01 g/kg/day	0.47 (0.37, 0.56) < 0.0001
Fat > 1.07 g/kg/day	−0.27 (−0.46, −0.07) 0.0080	Fat > 1.01 g/kg/day	−0.09 (−0.40, 0.22) 0. 5753
log likelihood test	<0.001	log likelihood test	0.002
**Physical activity**		**Physical activity**	
High		High	
Inflection point	0.45	Inflection point	0.44
Fat < 0.45 g/kg/day	0.29 (0.12, 0.46) 0.0007	Fat < 0.44 g/kg/day	1.30 (0.78, 1.83) < 0.0001
Fat > 0.45 g/kg/day	0.02 (−0.00, 0.03) 0.1025	Fat > 0.44 g/kg/day	0.20 (0.14, 0.25) < 0.0001
log likelihood test	0.001	log likelihood test	<0.001

All confounding factors were adjusted. The model is not adjusted for the stratification variable itself in the subgroup analysis.

### 3.3 Association of dietary fat intake with GSMAX_BMI_

In model 3, a positive correlation was observed between total fat intake and GSMAX_BMI_ [β 0.224, 95% CI:(0.192, 0.255)] (Table 2). Moreover, the consumption of dietary saturated fat [β 0.450, 95% CI:(0.372, 0.529)], monounsaturated fat [β 0.460, 95% CI:(0.382, 0.539)], polyunsaturated fat[β 0.466, 95% CI: (0.374, 0.558)], and total ω-3 fat [β 0.003, 95% CI:(0.002, 0.004)] exhibited positive associations with GSMAX_BMI_, while the intake of DHA [β−0.006, 95% CI:(−0.011, −0.000)], EPA [β−0.009, 95% CI:(−0.018, −0.000)], percentage of energy from fat [β−0.296, 95% CI:(−0.431, −0.161)] demonstrated negative associations (Table 2) when stratifying by gender, age, protein intake and physical activity, the association was significant between total fat intake and GSMAX_BMI_ (Figure 2). The trend analysis revealed a statistically significant association between the highest quartile of total fat intake and GSMAX_BMI_ (p for trend < 0.001), with the lowest quartile serving as the reference group (Figure 2).

**FIGURE 5:**
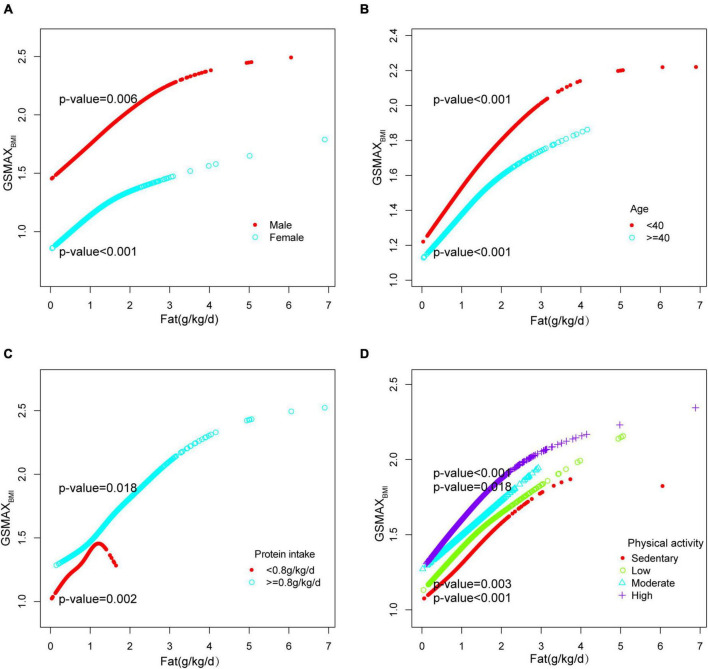
Stratified analysis for total fat intake (g/kg/d) and GSMAX_BMI_ dose–response relationship. **(A)** Stratified by sex **(B)** Stratified by age **(C)** Stratified by protein intake (g/kg/d). **(D)** Stratified by physical activity confounding factors were adjusted. The model was not adjusted for the stratification variable itself in the subgroup analysis.

Furthermore, in this study, we employed an adjusted smooth curve fitting technique to account for the non-linear correlation between total fat intake and GSMAX_BMI_ (Figure 3B), while also considering gender, age, protein intake, and physical activity as stratification factors (Figure 5). The results of our study indicated a noteworthy threshold (1.64 g/kg/day) at which a significant relationship between total fat intake and GSMAX_BMI_ was observed. Beyond this threshold, the rate of increase in GSMAX_BMI_ decreased significantly. GSMAX_BMI_ exhibited an increasing trend with total fat intake until reaching the inflection point (1.01 g/kg/day) among individuals with protein intake below 0.8 g/kg/d (Figure 5 and Table 3). Collectively, the relationship between total fat intake and GSMAX_BMI_ demonstrated an inverted U-shaped curve.

## 4 Discussion

The present analysis utilized data from the 2011 to 2014 NHANES survey, focusing on adults aged 20–59 years, to investigate the association between weight-adjusted daily fat intake and both muscle mass and muscle strength. Within our cross-sectional analysis of 5,356 participants, we found positive associations between total fat, saturated fatty acids, monounsaturated fatty acids, and polyunsaturated fatty acids with both muscle mass and muscle strength. Conversely, EPA, DHA, and the ratio of fat to total energy exhibited negative correlations with muscle strength as determined through multiple linear regression analysis.

A cross-sectional study conducted in Korea with 10,863 individuals aged 40 years and above identified low fat intake as a risk factor for muscle mass loss (23), which is consistent with our findings. Another Chinese cross-sectional study of 861 older people in three regions showed that abundant dietary protein intake and lower energy from fat were linked to better muscle mass and strength (24), also supporting our findings. Additionally, a longitudinal study conducted in Japan suggested that the dietary intake of short-chain fatty acids could potentially prevent the decline in muscle strength among community-dwelling older adults (16). However, a cross-sectional study of 5,412 participants aged 6–17 years who attended NHANES between 2011 and 2018 showed that low fat consumption may help prevent sarcopenic obesity (25). The 13-week trial in America found that dietary fat intake affected intramyocellular lipid content among healthy and aged individual (18). This had detrimental effects on muscle metabolism. Related studies (26–28) have shown the mechanism that adipocytes produce adipokines to increase leptin, chemerin, resistin, and decrease adiponectin, then increase tumor necrosis factor (TNF -α), interleukins (ILs), interferon (INF-γ), thereby creating a condition of low-grade inflammation. This is the condition that can result in dysfunction and apoptosis of the affected myocytes. This discrepancy may be attributed to variations in the age composition of the study population.

Additionally, multiple studies have demonstrated the beneficial effects of ω-3 and the unsaturated fat to saturated fat ratio on muscle health (29–32), potentially attributed to their antioxidant properties (33, 34). For instance, a longitudinal study conducted in Italy involving 159 elderly patients with diabetes, followed for a duration of 94 months, revealed a significant impact of high-fat intake on muscle health (35). Similarly, a cross-sectional analysis of elderly participants in the Cardiovascular Health Study yielded comparable results. However, the existing literature presents conflicting findings regarding the effects of DHA and EPA on muscle health, with some studies reporting positive outcomes (36–40) while others do not (41, 42). In contrast to these previous investigations, our study indicated that supplementation with EPA and DHA had detrimental effects on muscle health through multiple linear regression analysis. It needed further research.

In our study, we further examined quartiles of fat intake and found that higher levels within a specific range of fatty acid intake were associated with improved muscle mass and strength. Our stratified analysis also found no significant association between fat intake and muscle mass in men, which differs from the result of a cross-sectional study of 441 middle-aged Arab men (43). In the meantime, the results of our study indicated no significant correlation between dietary fat intake and muscle mass when protein intake exceeds 1.5 g/kg/day after adjusting for potential confounding variables. However, a cross-sectional study in China found an association between fat intake and muscle mass when protein intake above 1.7 g/kg/day (24), which requires further investigation.

In addition, our analysis using a saturated effect model and smooth curve fitting revealed an inverted U-shaped relationship between total fat intake and muscle mass, with a turning point of 1.88 g/kg/day. For males, the turning point was 2.22 g/kg/day. When protein intake was less than 0.8g/kg/d, there was a negative correlation between fat intake exceeding 1.07 g/kg/day and muscle mass; additionally, there was a negative correlation between fat intake exceeding 1.01 g/kg/d and muscle strength. It is worth noting that in populations with high physical activity levels, the inflection points were found to be only at 0.45 g/kg/d and 0.44 g/kg/d. Beyond these thresholds, there was a sharp decline in muscle mass and strength, accompanied by an increasing trend in fat intake. In conclusion, it is recommended that individuals between the ages of 20–59 limit their fat intake to less than 1.88 g/kg/day in order to prevent the decline of muscle mass and strength. However, this restriction may be less stringent for men and more stringent for individuals who experience protein deficiency or engage in high levels of physical activity.

To date, there is a scarcity of research examining the impact of fat consumption on muscle strength and mass, with a dearth of comprehensive investigations in this area and inconclusive findings. Furthermore, the majority of studies have primarily focused on older adults. However, it is crucial to acknowledge that muscle characteristics undergo changes early in life, thus emphasizing the significance of preventive measures in contemporary society. Consequently, we conducted a cross-sectional study encompassing individuals aged 20–59, which revealed a non-linear association between fat intake and muscle mass and strength, ultimately proposing an upper limit of 1.88 g/kg/d. Moreover, it was found that the upper limit for fat intake on a low-protein diet was 1.01 g/kg/day, and this limit was even lower for individuals with high physical activity levels. Meanwhile, a certain amount of fat intake is necessary.

This study is advantageous as it utilized data from a nationally representative sample, and the quality of this data was assessed using “gold standard” DXA. The study also examined protein intake per kg of body weight, as well as ALM_BMI_ and GSMAX_BMI_, as both continuous and categorical variables in order to gain a better understanding of the relationships between these variables. However, the applicability of cut-points for low lean mass derived from data in older adults to adults aged 20–59 is debatable. Nevertheless, the utilization of a continuous variable as the object of analysis helps mitigate some uncertainties. It is important to note that this cross-sectional study lacks the ability to establish causality between fatty acid depletion and muscle. Therefore, additional prospective clinical studies and fundamental research are necessary to substantiate these findings. Furthermore, no additional analyses were conducted to determine the smooth-curve fitting for saturated fat, unsaturated fat, and DHA. Hence, it is recommended that a more extensive prospective study be conducted in the future to gain a deeper comprehension of the causal connection between fatty acids and muscle.

## 5 Conclusion

The moderate dietary fat intake (less than 1.88 g/kg/day) can be beneficial for muscle mass and strength in adults aged 20–59. Furthermore, individuals with low protein intake or high physical activity levels may need to limit their fat intake more strictly for optimal muscle health, with a recommended maximum of 1.01 g/kg/day for those with low protein intake. Randomized clinical trials are necessary to confirm our observations.

## Data availability statement

The original contributions presented in this study are included in this article/supplementary material, further inquiries can be directed to the corresponding author.

## Ethics statement

The studies involving human participants were reviewed and approved by National Health and Nutrition Examination Survey, NCHS IRB/ERB Protocol Number: NHANES 2011–2012 (Protocol #2011–17); NHANES 2013–2014 (Continuation of Protocol #2011–17). The participants provided their written informed consent to participate in this study.

## Author contributions

SW: Data curation, Funding acquisition, Writing – original draft, Writing – review and editing, Formal Analysis. YZ: Data curation, Formal Analysis, Writing – original draft. DZ: Data curation, Formal Analysis, Writing – original draft. FW: Supervision, Writing – review and editing. WW: Supervision, Writing – review and editing. QW: Data curation, Formal Analysis, Writing – original draft. YB: Data curation, Formal Analysis, Writing – original draft. KY: Funding acquisition, Supervision, Validation, Writing – review and editing.
